# Lymphocytic Choriomeningitis—Emerging Trends of a Neglected Virus: A Narrative Review

**DOI:** 10.3390/tropicalmed6020088

**Published:** 2021-05-25

**Authors:** Tatjana Vilibic-Cavlek, Vladimir Savic, Thomas Ferenc, Anna Mrzljak, Ljubo Barbic, Maja Bogdanic, Vladimir Stevanovic, Irena Tabain, Ivana Ferencak, Snjezana Zidovec-Lepej

**Affiliations:** 1 Department of Virology, Croatian Institute of Public Health, 10000 Zagreb, Croatia; maja.bogdanic11@gmail.com (M.B.); irena.tabain@hzjz.hr (I.T.); ivana.ferencak@hzjz.hr (I.F.); 2 School of Medicine, University of Zagreb, 10000 Zagreb, Croatia; anna.mrzljak@gmail.com; 3 Laboratory for Virology and Serology, Poultry Center, Croatian Veterinary Institute, 10000 Zagreb, Croatia; v_savic@veinst.hr; 4 Clinical Department of Diagnostic and Interventional Radiology, Merkur University Hospital, 10000 Zagreb, Croatia; thomas.ferenc95@gmail.com; 5 Department of Gastroenterology and Hepatology, Clinical Hospital Center Zagreb, 10000 Zagreb, Croatia; 6 Department of Microbiology and Infectious Diseases with Clinic, Faculty of Veterinary Medicine, University of Zagreb, 10000 Zagreb, Croatia; ljubo.barbic@vef.hr (L.B.); vladostevanovic@gmail.com (V.S.); 7 Department of Immunological and Molecular Diagnostics, University Hospital for Infectious Diseases “Dr Fran Mihaljevic”, 10000 Zagreb, Croatia; szidovec@gmail.com

**Keywords:** lymphocytic choriomeningitis virus, epidemiology, pregnancy, transplant recipients

## Abstract

Lymphocytic choriomeningitis virus (LCMV) is a neglected rodent-borne zoonotic virus distributed worldwide. Since serologic assays are limited to several laboratories, the disease has been underreported, often making it difficult to determine incidence and seroprevalence rates. Although human clinical cases are rarely recorded, LCMV remains an important cause of meningitis in humans. In addition, a fatal donor-derived LCMV infection in several clusters of solid organ transplant recipients further highlighted a pathogenic potential and clinical significance of this virus. In the transplant populations, abnormalities of the central nervous system were also found, but were overshadowed by the systemic illness resembling the Lassa hemorrhagic fever. LCMV is also an emerging fetal teratogen. Hydrocephalus, periventricular calcifications and chorioretinitis are the predominant characteristics of congenital LCMV infection, occurring in 87.5% of cases. Mortality in congenitally infected children is about 35%, while 70% of them show long-term neurologic sequelae. Clinicians should be aware of the risks posed by LCMV and should consider the virus in the differential diagnosis of aseptic meningitis, especially in patients who reported contact with rodents. Furthermore, LCMV should be considered in infants and children with unexplained hydrocephalus, intracerebral calcifications and chorioretinitis. Despite intensive interdisciplinary research efforts, efficient antiviral therapy for LCMV infection is still not available.

## 1. Introduction

Lymphocytic choriomeningitis virus (LCMV) was isolated in 1933 by Armstrong and Lillie from a cerebrospinal fluid of a patient with meningoencephalitis detected during the St. Louis encephalitis outbreak (St. Louis, Missouri, USA). In 1935, Traub identified the house mouse (*Mus musculus*) as the natural reservoir host of the virus [1]. During the years that followed its discovery, LCMV was identified as one of the most common causes of aseptic meningitis in humans. Between 1953 and 1958, the virus was detected in 58 of 713 patients with neuroinvasive disease in the USA [2]. In addition, large outbreaks occurred in Germany, 1968–1971 (47 cases) [3], and USA, 1973–1974 (181 cases) [4]. Although LCMV is prevalent among mice in the Americas, Africa, Asia, and Europe, in more recent decades, human clinical cases are rarely recorded. Therefore, the disease has been underreported, often making it difficult to determine incidence and seroprevalence rates [5]. Two more recent studies have shown LCMV RNA in 5.1% of neuroinvasive cases in Iraq (2012–2013) [6] and LCMV antibodies in 5% patients with neurological symptoms in Finland (2013–2014) [7]. Congenital LCMV infection was initially recognized in England (1955) in a child born 12 days after maternal illness developed. The child died on the 12th day of life displaying signs of meningoencephalitis [8]. Further reports from Germany, Lithuania, and France in the 1970s have documented the association of intrauterine LCMV infection with the occurrence of spontaneous abortion as well as with hydrocephalus, intracranial calcifications, and chorioretinitis in newborns [9,10,11]. In 1993, the first case of congenital LCMV infection was reported in the USA [12]. The majority of congenitally infected children show long-term neurologic sequelae [13]. Identification of a fatal LCMV infection in several clusters of organ transplant recipients (2003–2013) has resulted in increased awareness on the emerging role and the pathogenic potential of LCMV. In these populations, CNS abnormalities were also found, but were overshadowed by the systemic illness resembling the Lassa hemorrhagic fever [14,15].

This review focuses on the emerging trends in the epidemiology, clinical characteristics, molecular epidemiology, and diagnosis of LCMV infection, a neglected rodent-borne viral zoonosis. A literature search was conducted in the following databases: Web of Science, PubMed, Medline, and Scopus along with hand-searching references of key articles and a ResearchGate and Google search with no limitations placed on the year of publication and language restriction. Books and dissertations were also included. Keywords searched were: lymphocytic choriomeningitis virus, epidemiology, seroprevalence, humans, rodents, congenital infection, TORCH, transplant, molecular epidemiology, diagnosis, therapy, immunology, vaccine. Once a comprehensive list of abstracts were retrieved and reviewed, studies appearing to meet inclusion criteria were reviewed in full.

## 2. Structure and Genome Organization of LCMV 

LCMV is an enveloped single-stranded negative sense RNA virus that belongs to the *Arenaviridae* family, genus *Mammarenavirus*. The virus is round, oval, or pleomorphic, 110 to 130 nm in diameter. During morphogenesis, sandy-appearing granules resembling ribosomes are found within the virus on the electron microscopy, giving arenaviruses their name (Latin *arena* = sand). The helical nucleocapsid contains an RNA genome consisting of two ambisense RNA segments (S and L) that encode four viral proteins: nucleoprotein (NP), envelope precursor glycoprotein (GPC), which is cleaved into two subunits (GP1 and GP2), matrix zinc-binding (Z) protein and the large (L) RNA-dependent RNA polymerase (RdRp). A stable signal peptide (SSP) cleaved during GPC synthesis is also in the virion spike. NP is the arenaviral major structural protein, essential for both transcription and replication. Antigens on GP1 are involved in virus neutralization [16].

There are several strains of LCMV that exhibit distinct patterns of tissue tropism. LCMV Armstrong is the original virus strain isolated from the brain by Charles Armstrong in 1933. The Traub strain was isolated from a laboratory colony of persistently infected mice in 1935 [1] and the WE strain was isolated in 1936 from the infected patient after exposure to persistently infected mice. The WE strain is viscerotropic in mice but is more frequently associated with aseptic meningitis in primates than is neurotropic Armstrong strain [17]. More than 30 additional strains have since been isolated from rodents and humans in the USA, Europe, and Japan [18,19]. Many variants of these strains also exist. LCMV Clone 13 is a variant of the Armstrong viral strain, isolated from the spleen and is consequently tropic for visceral organs [19] and the “Docile” is a derivative of the WE strain [20]. 

LCMV replicates in a wide variety of cell types. During infection, the viruses attach to cell surface receptors and are internalized by endocytosis. Transcription and replication is confined to the cytoplasm of the infected cells. The S RNA encodes in the negative sense a NP and in the positive sense a GPC, which is post-translationally cleaved into GP1 and GP2. The L RNA encodes in the negative sense an RdRp, and in the positive sense a Z protein, which binds to the RNP complex. The viruses bud from the plasma membrane, incorporating host lipids into the virus membrane [16].

## 3. Epidemiology of LCMV

The natural rodent host and reservoir for LCMV are the common house mice (*M. musculus, Mus domesticus*). Mice infected in the intrauterine period fail to mount an immune response and develop chronic, asymptomatic, life-long infection resulting in shedding of large quantities of virus in nasal secretions, saliva, milk, semen, urine, and feces [21]. Human infections occur through mucosal exposure to aerosols contaminated with rodent excreta, direct contact with rodents or through rodent bites [16]. Pet mice and hamsters have also been identified as sources of infection. Several outbreaks of LCMV infection in humans have been linked to exposure to persistently infected hamsters [22,23]. Human-to-human transmission is not documented, except through organ transplantation [14,15] and vertical transmission from infected pregnant women to fetus [8]. 

LCMV acquired from mice has also caused a highly fatal hepatitis in captive Callitrichid primates. Callitrichid hepatitis occurs as sporadic outbreaks among many species of marmosets and tamarins [24].

**Figure 1:**
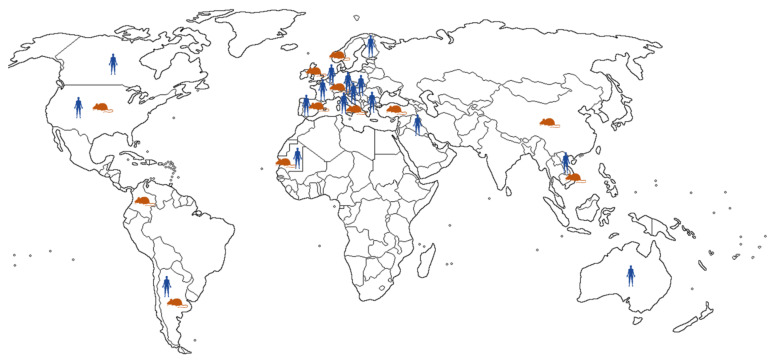
Geographic distribution of LCMV infections reported in humans and rodents.

Geographic distribution of LCMV is presented in Figure 1.

**Figure 2:**
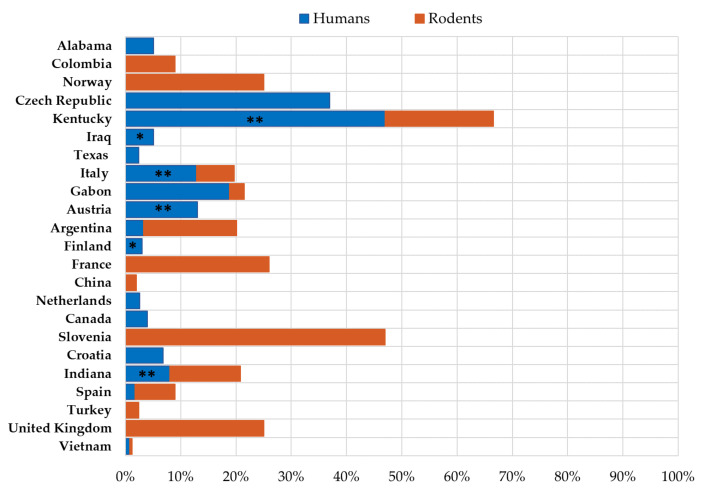
Seroprevalence of LCMV in humans and rodents. (* patients with neuroinvasive disease, ** professionally exposed persons).

The seroprevalence of LCMV in humans and rodent reservoirs differs greatly between regions (Figure 2).

### 3.1. LCMV Prevalence in Humans

Because of the cosmopolitan distribution of its reservoirs, LCMV most likely circulates worldwide. However, since many LCMV infections are asymptomatic and mild, the true prevalence is unknown. 

In 1950s, LCMV was detected in 8% of hospitalized patients with neuroinvasive disease, especially during winter months when mice move indoors [25]. A more recently published study from Finland (2013–2014) has found that 5.0% of patients with neuroinvasive disease tested positive for LCMV IgG antibodies. Seropositivity was equally distributed between female and male patients, with the highest rate being in 5 to 10-year-olds (16%) [7]. Additionally, LCMV RNA was detected in 5.1% of cerebrospinal fluid (CSF) samples from patients with neuroinvasive disease in southern Iraq (Nasiriyah region, 2012–2013) [6]. Another study from the same region (2012–2016) analyzed the prevalence of LCMV in patients with fever and neurologic manifestations and healthy persons. The seroprevalence was 12.2% in the healthy control group and 7% in the acute febrile patients. Moreover, two CSF samples from patients with fever and neurologic symptoms were positive for LCMV RNA [26]. 

Seroprevalence studies conducted in the general population have revealed that up to 15% of the population is LCMV seropositive [6,7,27,28,29,30]. In the 1990s, the reported seroprevalence rates were found to be 2.3% (1.54–6.06%) in residents of Argentina [31], 2.4% in Texas (San Antonio) [32], 4% in Canada (Nova Scotia) [33], and 4.3–5.1% in Alabama (Birmingham) [32]. Two studies from 2000s showed a seropositivity of 1.7% in Spain [27] and 3.3% in Argentina [28]. A more recent study (2015–2017) showed a high LCMV seropositivity of 21.5% if the Gabonese residents with significantly higher seropositivity in male (29.2% vs. 17.8%) and adult (36.1% vs. 6.9%) populations [34].

There are very few data on the seroprevalence of LCMV in pregnant women. In Argentina, 1.6% of pregnant women were found to be seropositive [28]. In addition, 3.9% of Croatian pregnant women showed LCMV antibodies [35]. However, IgM antibodies were not detected in both studies, suggesting previous LCMV infection only. 

A very low LCMV IgG prevalence rate of 0.33% has been documented among blood donors in Marseilles, southeastern France in 2007 and blood donors from New York in 2009 (0.33 and 0.2%, respectively) [36,37].

Several studies showed higher LCMV seropositivity in persons exposed to rodents. An Austrian study found that 13% of employees of the zoological garden of Vienna were LCMV seropositive [38]. A study from Italy conducted among forest workers in the Province of Trento showed an increase in the LCMV seroprevalence from 2.5% in 2002 [29] to 7% in 2015, but no risk factors such as occupation and many outdoor activities (gardening, having a woodshed, having a pet rodent and collecting mushrooms) were significantly associated with this increase. Additionally, seroprevalence was high in hunters (12.9%) [39]. In 2012, 8 to 47% of employees of rodent breeding facilities in the USA (Indiana and Kentucky) tested LCMV seropositive [40]. However, persons living in farming communities in the Mekong Delta of Vietnam (2012–2013) showed a very low seropositivity (0.8%) [30].

Very high LCMV seroprevalence rates have been reported in some hyperendemic areas. A seropositivity of 37.5% was reported among inhabitants of Bratislava, Slovakia in 1999 [41]. In addition, in 2006, LCMV antibodies were detected in 36% of the rural population of the Vir, a small island at the Croatian littoral, which is an endemic region for murine typhus. Seropositivity was high in all age groups ranging from 32 to 40% [42]. However, more recent data (2017–2018) showed an overall seroprevalence of 6.8%, ranging from 3.9% in non-exposed populations to 9.8% in professionally exposed persons (forestry workers, hunters, persons in frequent contacts with rodents) from continental Croatian regions [35,43].

### 3.2. LCMV Prevalence in Rodents 

Epidemiological studies have shown that substantial proportions of wild mice are infected with LCMV in some regions, in both urban and rural settings (Figure 2).

In 1992–1993, LCMV antibodies were detected in 2.9% of wild mice (*M. musculus*) captured at 35 different sites in China [44]. During 1998–2003, the prevalence of LCMV antibodies in *M. musculus* was reported to be from 6.9 to 20.1% in urban areas of Argentina [31]. Furthermore, LCMV antibodies were detected in 25% of Norway rats (*Rattus norvegicus*) from the United Kingdom farms [45]. In addition, 5.6% of wild rodents (6.1% *Apodemus flavicollis*, 3.3% *Clethrionomys glareolus* and 14.3% *Microtus arvalis*) tested during 2000–2003 in Trentino, Italy, were LCMV seropositive [29]. A similar seroprevalence rate (6.8%) was found in wild rodents (*A. flavicollis*, *Myodes glareolus*, *M. arvalis*) in another study from northern Italy [46]. In 2004, LCMV antibodies were detected in 2.4% of rodents trapped in northeastern and western Turkey [47]. In Spain, 9% of mice (*M. musculus*, *Mus spretus*) caught in households and parks tested LCMV IgG positive with the seroprevalence varying from 7.5% in the northwest to 10.6% in the southeast. None of the rats (*Rattus rattus*) were LCMV seropositive [27]. In the municipality Sincelejo, Colombia, the seroprevalence of LCMV among mice was 10% [48]. From 2011 to 2013, the presence of LCMV antibodies was demonstrated in 24.93% of rodents collected in the Istrian Penninsula. The highest seroprevalence was found in commensal *M. musculus* (47.37%), followed by 11.53%, 19.04%, and 25% prevalence in *A. agrarius*, *A. flavicolis* and *A. sylvaticus*, respectively. The prevalence was the highest (53.33%) in locations with illegal waste sites and high anthropogenic influence, while it was significantly lower in rodents sampled from natural habitats [49]. In 2012, trapped rodents from three breeding facilities in the USA (Indiana, Kentucky) were tested for the presence of LCMV. In Indiana, 20.8% of mice had LCMV IgG antibodies, and 0.7% had detectable RNA. In Kentucky, 66% of mice were LCMV seropositive. Rats were not found to be infected [40]. In contrast, only 0.4% of rats purchased in wet markets or trapped in peridomestic and forest habitats in the Mekong Delta in southern Vietnam (2012–2013) tested LCMV seropositive [30]. A recently published study analyzed the prevalence of LCMV in rodents from Gabon captured during 2019–2020. Since bushmeat is widely consumed in Gabon, bushmeat samples were also included for the investigation. LCMV RNA was detected in a rodent (*Lophuromys sikopusi*) and a porcupine (*Atherus africanus*). House mice (*Mus minutoides, Praomys missonei*) and shrews (*Crocidura goliath*) were positive for LCMV antibodies with seroprevalence rates of 2.72% and 2.52%, respectively. LCMV IgG antibodies were not detected in any bushmeat samples [34].

### 3.3. Molecular Epidemiology of LCMV 

**Figure 3:**
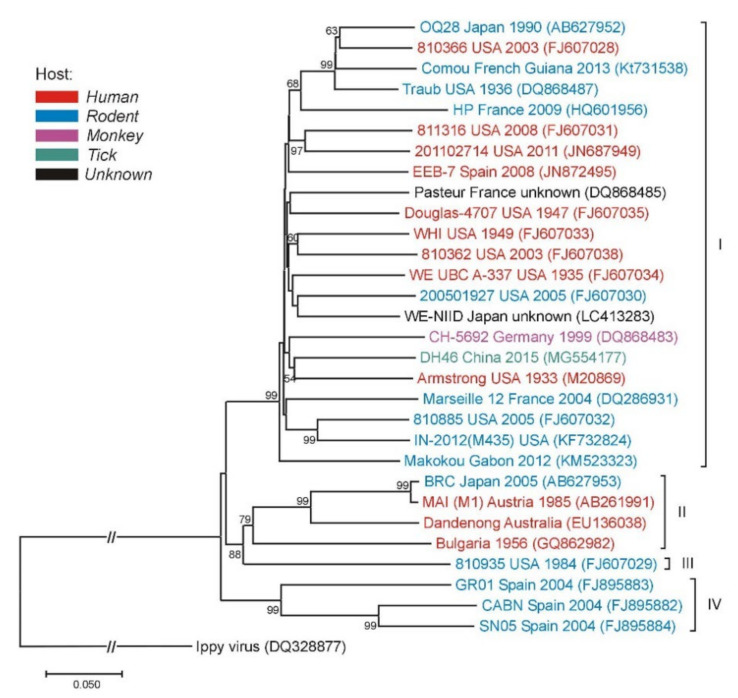
Phylogenetic neighbor-joining analysis of the lymphocytic choriomeningitis virus (LCMV) based on the small (S) gene segment. The tree is rooted with the Ippy virus. Strain/isolate designations, countries of origins, isolation/detection years and GenBank accession numbers are indicated at the branches. LCMV genetic lineages are indicated on the right. Supporting (≥50%) bootstrap values of 1000 replicates are displayed at the nodes. Horizontal distances are proportional to genetic distance. Scale bar indicates nucleotide substitutions per site. The interrupted branches, indicated by double slashes, were shortened by 50% for better graphic representation.

Phylogenetic analyses of LCMV strains collected from a variety of geographic and temporal sources showed this virus to be highly diverse. Analysis of the S segment separates the four major lineages. Most of the LCMV strains clustered within lineage I, which contains all the US strains, with the exception of the virus isolate from Georgia in 1984, the only member of lineage III. Lineage I includes the classic laboratory Armstrong and WE strains, as well as strains from France, Germany, and Slovakia. Lineage II appeared to only contain viruses from Europe. Lineage IV contains viruses isolated from wild-caught wood mice in Spain (Figure 3) [50]. 

Although the lineages I–III have all been associated with severe human disease, recent data on the genetic characterization of human LCMV infection are scarce. Two LCMV Iraqi isolates from neuroinvasive cases belonged to the lineage I [6]. The LCMV strain related to genetic lineage I composed of strains inducing severe disease in humans was detected in a house mouse (*M. musculus*) in French Guiana [51]. In addition, LCMV lineage I was detected in mice tested at the rodent breeding facility in Indiana [40]. A novel strain of LCMV (HP65-2009) was found in two *M. musculus domesticus* rodents trapped in the southwestern France. Genetic and phylogenetic analyses comparing LCMV HP65-2009 with 26 other LCMV strains showed that it represents a novel highly-divergent strain within the group of *M. musculus*-associated LCMV [52]. In 2015, LCMV was isolated from ticks in northeastern China. Phylogenetic analysis grouped the tick LCMV strains together with the lineage I strains, but in an isolated cluster with a high bootstrap value [53].

## 4. Clinical Characteristics of LCMV Infection

Acquired LCMV infection in immunocompetent persons may be asymptomatic (one third of infected persons) or presented as a non-specific, self-limited febrile disease. However, the illness can progress to meningitis or meningoencephalitis. Most cases recovered fully within one to three weeks [54]. In immunocompromised patients, such as organ transplant recipients, infection can result in multisystem organ failure resembling the Lassa hemorrhagic fever with a hepatitis as a prominent feature and a very high fatality rate. Pregnant women who acquired the LCMV infection during pregnancy may transmit the virus transplacentally to the fetus. Infection causes risk for miscarriage, intrauterine fetal death as well as severe CNS or ocular malformations (Table 1).

### 4.1. LCMV Infection in Immunocompetent Individuals

About one-third of children and adults who acquire LCMV are asymptomatic. Of the remaining two-thirds, about half present with non-specific febrile disease while the remaining have central nervous system disease. Symptoms appear 6 to 20 days after exposure. Acquired LCMV infection typically shows a biphasic course. The initial symptoms are non-specific and include fever, headache, malaise, myalgia, anorexia, nausea, and vomiting. After a temporary improvement, a second phase occurs presenting with the CNS symptoms (headache, photophobia, vomiting, and nuchal rigidity) [54]. Rarely, the course of the disease may be severe, including encephalitis, hydrocephalus and transverse myelitis [62]. Additionally, some rare non-neurologic complications of LCMV infection include pneumonitis, myocarditis, orchitis, and parotitis. Acquired LCMV infection is usually non-fatal with recovery without sequelae in most cases. The overall mortality rate is less than 1% [54].

During the initial febrile phase, laboratory abnormalities may include leukopenia, thrombocytopenia and mild elevations of liver enzymes. The hallmark laboratory abnormality during the CNS phase of the disease is a CSF pleocytosis which exceeds those typically observed in majority of viral meningitis. Hypoglycorrhachia and mild elevations of CSF protein level may also occur [54].

### 4.2. LCMV Infection in Immunocompromised Patients/Transplantation Associated LCMV Infection

**Figure 4:**
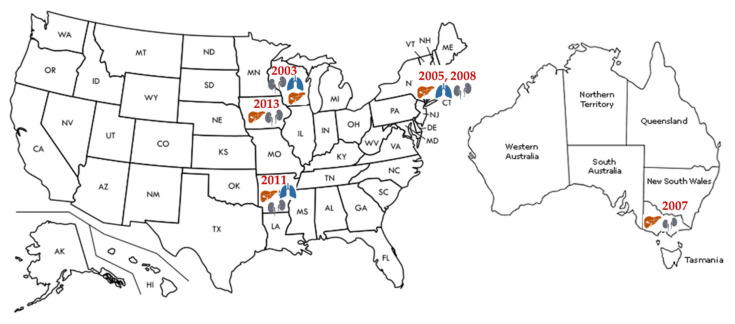
Transplantation associated LCMV infection.

Immunocompromised persons are at increased risk for developing severe LCMV disease. LCMV in transplant recipients has been mainly described in the setting of donor-derived infections. Several clusters of LCMV transmission with a total of 21 cases were reported mainly in kidney but also liver and lung transplant recipients [15,55,56,57,58,59] as recently reviewed by the Infectious Diseases Community of Practice of the American Society of Transplantation (Figure 4) [63]. However, cornea transplant recipients did not develop LCMV infection after transplantation [15,55]. A common donor in each cluster transmitted the infection to multiple recipients. The disease clinically presented early after the transplant (within several weeks), with fever, leukocytosis, abdominal pain, hepatitis, thrombocytopenia, coagulopathy, altered mental status, seizures, graft dysfunction, diarrhea, or rash, progressing quickly to a multisystem organ failure with a high mortality of 71%. Only one donor [55] had confirmed contact with a pet hamster, whereas in all other cases, the exposure to rodents could not be identified. Several donors died of intracranial hemorrhage without symptoms of LCMV infection. So far, only one case reported the acquisition of the infection several years after the transplant. A kidney transplant recipient developed LCMV meningoencephalitis complicated by hydrocephalus after contact with mice excreta [64]. The patient survived but required ventriculoperitoneal shunt placement. The common feature in immunocompromised populations is a delayed LCMV seroconversion, which additionally postpones the diagnosis and contributes to high mortality. The challenges of LCMV diagnostics and treatment have been recognized in the Guidelines from the American Society of Transplantation Infectious Diseases Community of Practice [63], addressing the need to increase the awareness and suspicion for this underdiagnosed disease. Unfortunately, potential organ donors screening for LCMV is still not recommended due to the low incidence of infection and limitations of current diagnostics. 

### 4.3. Congenital LCMV Infection 

LCMV infection in the early first-trimester is associated with an increased risk of spontaneous abortion [65]. Hydrocephalus, periventricular calcifications, and chorioretinitis are the predominant characteristics of congenital LCMV infection, occurring in 87.5% of cases [54]. Neonatal meningitis can also occur. Transplacental LCMV transmission and fetal infection presumably occurs during maternal viremia, primarily during the first and second trimesters, however intra-partum transmission could not be excluded [66]. Mortality among infants diagnosed with congenital LCMV infection is approximately 35%, and two thirds of survivors show long-term neurologic sequelae (microcephaly, mental retardation, seizures, visual impairment) [13]. Unlike the other classic congenital TORCH infections, the virus exhibits a strong neurotropism. Few infants congenitally infected with LCMV have organ involvement outside the central nervous system and rarely show signs of systemic infection such as hepatosplenomegally or rash [67]. Animal models have demonstrated that LCMV exhibits a very strong tropism for neuroblasts. The presence of mitotically active neuroblasts in the periventricular region of the fetal human brain explains the distribution of periventricular calcifications in congenital LCMV infection. In addition, it has been demonstrated that LCMV infection disturbs the migration of neurons, explaining gyral malformations in congenitally infected children [60,61].

Although congenital LCMV infection was believed to be rare (less than 100 cases reported worldwide so far) (Table 2), a greater availability of diagnostic tests has contributed to its better recognition. However, the true prevalence is unknown.

Differential diagnosis of congenital LCMV infection should include some other major TORCH pathogens such as cytomegalovirus (CMV), *Toxoplasma gondii*, rubella virus, herpes simplex virus, *Treponema pallidum,* and parvovirus B-19 [65]. CMV infection and toxoplasmosis may be particularly difficult to differentiate from LCMV, since all these infections can cause microcephaly, intracranial calcifications, and chorioretinitis. However, symptomatic neonatal CMV infections are generally associated with hepatosplenomegaly, which is rarely noted in children with congenital LCMV infections. Congenital rubella syndrome (CRS) is associated with heart disease, cataracts and deafness, all of which have been extremely uncommon in congenital LCMV infection. Additionally, CRS and syphilis are characterized by salt-and-pepper retinopathy not found in LCMV-infected infants. Furthermore, the bone and hepatic abnormalities that are characteristics of congenital syphilis have been virtually absent in congenital LCMV infection [61]. Congenital toxoplasmosis remains the major problem in differential diagnosis since chorioretinitis is the most common finding in both diseases. Intracranial calcifications are also very common in toxoplasmosis and LCMV. However, in congenital toxoplasmosis, diffuse intracerebral calcifications are usually present, in contrast to LCMV infection, which is characterized by periventricular calcifications [65].

## 5. Diagnosis of LCMV Infection

LCMV can be isolated from the blood and nasopharyngeal secretions early in the course of the disease or from the CSF in patients with meningitis. Virus titers in the CSF are lower and present for a shorter time period [84]. The virus can be grown in a variety of cell lines including BHK-21 (baby hamster kidney), L-929 (mouse fibroblasts) and Vero cells (green monkey kidney). A diagnosis can also be made by the intracerebral inoculation of blood or CSF into newborn mice. Many LCMV isolates produce a characteristic convulsive disease within 5 to 7 days, which is nearly pathognomonic. Brains from dead mice may be subjected to immunofluorescence assay (IFA) or immunohistochemical staining to obtain presumptive identification or for confirmatory testing by a reverse transcriptase-polymerase chain reaction assay (RT-PCR) [16]. A RT-PCR has also been developed for the detection of LCMV RNA in blood and CSF. The highly sensitive assays target the GPC and N genes [84,85]. The observed detection limits in plaque-forming units (PFU) of four LCMV isolates tested were ≤10 PFU/mL (Arm53b and WE54) and 1 PFU/mL (Traub and E350), respectively [85]. Serology is the most commonly used method for diagnosis of LCMV. Enzyme-linked immunoassay (EIA) and IFA can detect IgM and IgG antibodies, however these tests are limited to several laboratories. Since there are very few commercially available tests, several in-house EIA and IFA methods were developed with different sensitivity and specificity. In addition, there are many methods for determination of the EIA threshold. EIA with recombinant LCMV NP as antigen has been reported by several groups [86,87]. Comparing EIA and IFA, it seems that EIA is more sensitive. Moreover, some authors showed that the IFA method was accompanied by high background, which was significantly reduced in EIA [87]. It has been noted that IgG titers that have been measured by EIA persist longer than those obtained by IFA [61]. In contrast, the other authors found that results of EIA were in complete agreement with those of IFA [86]. Acute and convalescent serum samples can be tested for increase in antibody titers, whereas detection of specific IgM in blood and CSF is diagnostic [84].

For congenitally infected children, the diagnosis is more complicated. The majority of infants born with congenital LCMV infection no longer harbor the infectious virus at the time of birth. The diagnosis in these cases should be confirmed serologically. Trans-placentally transferred maternal IgG antibodies further complicate the diagnosis. For this reason, the serologic tests for LCMV should include both IgM and IgG titers on both maternal and infant serum samples [54].

In the transplant population, diagnosis of LCMV requires a combination of testing modalities, including detection of LCMV-specific IgM/IgG in CSF and serum and detection of LCMV by RT-PCR or virus isolation from CSF, serum, and tissues. Antigen detection using immunohistochemical staining in tissue specimens can also be helpful in cases of negative serology. Testing of serum and CSF by serology and RT-PCR is recommended to improve diagnostic yield [63]. Additionally, next-generation sequencing has been used retrospectively for the donor-derived LCMV infection [56].

## 6. Therapy of LCMV Infection 

Antiviral therapeutic options in human LCMV infection are currently exceptionally limited. The majority of research efforts on antiviral therapy in human LCMV infections are focused on re-purposing antiviral drugs approved for the treatment of other infectious diseases. 

The first antiviral drug repurposed for the treatment of transplantation-associated LCMV infection in humans was ribavirin, a guanosine analogue. Ribavirin is a broad-spectrum antiviral drug with complex mechanisms of action including direct inhibition of RdRp, induction of mutagenesis as well as inhibition of inosine monophosphate dehydrogenase leading to the depletion of guanosine triphosphate that has been used as a therapeutic option in a number of infectious diseases. In addition, ribavirin is a potent immunomodulatory drug that supports the differentiation of naive CD4+ T-cells towards a Th1-type cytokine responses that enhance antiviral immunity [88].

Fisher et al. (2006) described epidemiological, clinical and laboratory features of patients from the two clusters of solid-organ transplant-transmitted LCMV infections that occurred in 2003 and 2005 in the USA [55]. Seven of eight transplant recipients died in the period between days 9 and 76 after transplantation, apart from one kidney recipient (2005 cluster), who was treated with ribavirin and underwent reduction of immunosuppressive therapy levels, survived. The patient was treated with intravenous and oral ribavirin for 37 days until negative LCMV RT-PCR and immunohistochemistry results of renal biopsy specimens accompanied with IgM seronegativity, 67 days after transplantation [55]. However, other studies including a fourth USA cluster of transplant-associated LCMV infections showed that two of four recipients survived without ribavirin therapy [15]. Therefore, the evidence on the usefulness of ribavirin in human LCMV infections should be carefully evaluated. 

The antiviral drug favipravir (pyrazinecarboxamide derivative) inhibits the activity of RdRp of various RNA viruses. In addition to being approved for clinical use in influenza in Japan, it is currently used and evaluated in clinical trials for the therapy of COVID-19 [89]. In vivo experiments in a mouse model of acute disseminated LCMV infection and hemorrhagic disease showed excellent antiviral efficacy of favipravir. Early administration of favipravir in New Zealand black (NZB) mice infected with a low-dose of LCMV-Cl13 resulted in a complete protection from mortality and inhibition of viral replication to undetectable levels in the majority of animals [90]. These results suggested that favipravir could be considered as a possible antiviral therapeutic option in human LCMV infection as well. 

Umifenovir (arbidol) is an indolyc carboxylic acid that inhibits various stages of viral replication cycle by interaction with viral proteins and lipid components of the virion [91]. It is used for the prevention and treatment of influenza virus infection and is currently being evaluated in clinical trials as a COVID-19 antiviral drug. Herring et al. (2021) showed that umifenovir inhibits the replication of several arenaviruses including LCMV in vitro, suggesting that this drug might represent a possible therapeutic strategy in arenavirus infections [92]. 

Due to the limited antiviral strategies in LCMV infection, a number of studies evaluated the possible efficacy of various anti-LCMV antiviral compounds in vitro. Wan et al. (2020) performed a large-scale screening of the library of 63 FDA-approved drugs by using a recombinant LCMV-P2A-eGFP replication model in Vero and BHK-21 cells. A total of five drugs inhibited LCMV replication in a dose-dependent manner at different stages of the replication cycle: (1) benidipine hydrochloride inhibited membrane fusion and acted as an entry inhibitor, (2) mycophenolic acid, lapatinib, and dabrafenib inhibited viral replication and (3) clofazimine exhibited both mechanisms of action [93]. Possible efficiency of these compounds in animal models of LCMV infection as well as in clinical research remains to be determined. In addition, Kim et al. (2019) performed an extensive screening of 11,968 compounds in the ReFRAME (Repurposing, Focused Rescue and Accelerated Medchem) library and identified 10 anti-LCMV compounds inhibiting different steps in the replication cycle that represent promising candidates for further pre-clinical evaluation [94].

State-of-the art strategies of drug design have recently been applied in LCMV antiviral research as well. Bösch et al. (2020) showed that landornamide A, an ornithine-containing ribosomal peptide discovered via genome mining, inhibits LCMV infection in mouse cells in vitro. Landornamide A is the product of the silent osp gene cluster from *Kamptonema* sp. [95].

Despite intensive interdisciplinary research efforts, efficient antiviral therapy in human LCMV infection, particularly in transplanted patients, is currently not available.

Encouraging data regarding a possible therapeutic strategy comes from a related field, e.g., the treatment of Lassa virus infection. Mire et al. (2017) reported that combinations of human monoclonal antibodies (huMAbs) specific for glycoproteins of Lassa virus clades I-VI protect cynomologus macaques providing a 100% rescue, even when treatment is initiated at advanced stages of disease [96]. Monoclonal antibodies were derived from mature B-lymphocytes isolated from convalescent donors and were specifically selected to confer high level of protection from lethal Lassa fever in animal models. Several of these huMAbs (for example 37.2D and 12.1F) cross-react with LCMV glycoprotein complex in vitro, suggesting that further studies specifically focusing on monoclonal-antibody based treatment of LCMV are warranted [97,98]. 

## 7. LCMV Infection in Mice as an Immunological Model

The LCMV mouse model is a fundamental in vivo research tool in immunology that played an important role in understanding the key principles of both innate and specific immunity including immunological restriction mediated by Major Histocompatibility Complex (MHC), T-cell mediated lysis of virus-infected cells, perforin-based cytotoxicity, mechanisms of T-cell exhaustion in chronic viral infections, development of T-cell memory, contribution of immune responses to the pathogenesis of infectious diseases, and the key role of innate immunity [99,100]. The majority of experimental work in the LCMV mouse model is based on the use of six viral strains: Armstrong (LCMV-Arm 53b), Clone-13, Traub, WE, Aggressive, and Docile [101]. Important differences in the virology and pathogenicity of LCMV laboratory strains enabled their application in studies of different types of infection (acute vs. chronic) and the host’s antiviral responses. A slowly-replicating LCMV-Arm 53b strain is used as a model of acute infection that leads to a spontaneous viral clearance within two weeks but is also important in studies of lethal lymphocytic choriomeningitis that occurs following intracranial infection. LCMV clone 13 (LCMV-cl13) replicates faster than LCMV-Arm 53b and, subsequent to high-dose intravenous challenge, causes a persistent infection with a detectable viremia for up to 90 days. Interestingly, the two LCMV strains differ in only three aminoacids (5 of 10,600 nucleotides) showing that small numbers of mutations leading to very few aminoacid changes in the selected positions of key viral proteins can lead to important changes in the biology of viruses [99,100]. More recent studies on innate and specific immune responses in LCMV infection provided a new perspective on molecular mechanisms of immune responses with particular emphasis on recognition of LCMV and initiation of innate immune responses including type I IFNs and NK-cells. In addition, LCMV model made an important contribution in studies on the mechanisms of specific immunity, particularly thymic depletion in viral infection, modulation of Treg compartment, metabolic changes associated with chronic viral infection and mechanisms of indirect antibody-mediated protection (summarized in Table 3) [102,103,104,105,106,107,108]. 

## 8. LCMV in Vaccine Research

In addition to an important role as a research model in immunology, reverse genetically-engineered recombinant LCMV (rLCMV) is an important candidate in the development of vector-based vaccines. Krolik et al. (2021) recently reported results of safety and efficacy analysis of a non-replicating rLCMV vector expressing ovalbumin as a model antigen in immunocompromised Ifnar−/− mice that lacks a functional type I IFN receptor. Immunization of Ifnar−/− mice induced differentiation of multifunctional cytotoxic CD8+ T-cells and memory T-cells, leading to the clearance of rLCMV-ovalbumin vector within 7 days post-vaccination [109]. An excellent safety profile of the rLCMV viral vector derived from Clone 13 in combination with maintained efficacy in immunocompromised hosts suggest that non-replicating rLCMV-based vectors represent a promising candidate for vaccine development. 

Replicating LCMV-based vectors have been evaluated as possible therapeutic cancer vaccines aimed at inducing antitumor T-cell mediated immunity and subsequent long-term tumor control. Schmidt et al. (2020) constructed a new vaccine TT1-E7E6 based on replicating attenuated LCMV encoding a non-oncogenic version of oncoproteins E7 and E6 of human papillomavirus type 16 (HPV-16). Evaluation of TT1-E7E6 in a mouse model showed vector clearance, induction of CD8+ T-cells specific for HPV-16 and tumor control, suggesting that the LCMV-based TT1-E7E6 vaccine might represent an excellent candidate for the immunotherapy of HPV-16-positive cancers [110].

## 9. Conclusions

Although the proportion of meningitis cases attributed to LCMV has declined, at least in part due to the unavailability of commercially available serologic assays, this virus remains an important cause of meningitis in humans. However, clinical interest for the disease is low, and LCMV has been rarely considered. Fatal LCMV infection in several clusters of solid organ transplant recipients who received transplants from donors who died of apparent non-infectious etiologies further highlighted a pathogenic potential and clinical significance of this neglected human pathogen. Additionally, LCMV should be considered an emerging obstetric teratogen. Although only 82 cases of congenital LCMV infection have been reported so far, obstetricians should be aware of an emerging role of LCMV as a TORCH agent that can disturb maternal, fetal, and neonatal health.

## Figures and Tables

**Table 1:** Clinical symptoms of LCMV infection.

Population	Clinical Symptoms	Outcome	Reference
Immunocompetent individuals	Asymptomatic infection, flu-like disease, aseptic meningitis/meningoencephalitis	Recovery in most cases; mortality < 1%	[54]
Organ transplant recipients	Fatal hemorrhagic fever-like disease	Mortality > 70%	[15,55,56,57,58,59]
Pregnant women	Asymptomatic infection, flu-like disease, aseptic meningitis/meningoencephalitis; symptoms may be severe	Depending on the time of infection: spontaneous abortion, congenital LCMV infection	[60,61]
Newborns (congenital infection)	Hydrocephalus, periventricular calcifications, chorioretinitis	Mortality ~ 35%; persistent neurologic sequelae ~ 70%	

**Table 2:** Congenital LCMV infection.

Country/Year	N Cases	Clinical Features	CT/MRI Imaging	Reference
England 1955	1	Convulsion, opisthotonos, subarachnoid and intracerebral haemorrhage, petechiae, ventriculomegaly	ND *	[8]
Germany 1974, 1999	8 (2-twins)	Hydrocephalus, microcephaly, intracranial calcifications, chorioretinitis, chorioretinal scars, blindness, conjuctivitis, developmental delay, myocarditis, congestive heart failure, psychomotor retardation, meningitis	ND	[9,68]
Lithuania 1976, 1981	22	Hydrocephalus, microcephaly, spastic tetraparesis, epilepsy-like attacks, chorioretinal degeneration, optic disc subatrophy, microphtalmy, cataract, psychomotor retardation	ND	[10,69]
France 1978, 2000, 2009, 2017	6 (2-twins)	Hydrocephalus, ventriculomegaly, microcephaly, periventricular calcifications, chorioretinitis chorioretinal scars, fetal hydrops, hepatosplenomegaly, cardiomegaly, ascites, pericardial and pleural effusion	MRI: normal (2 cases)	[11,70,71,72]
USA 1993, 1995, 1996, 1997, 2000, 2002, 2003, 2006, 2007, 2013, 2014, 2018	45 (2-twins)	Hydrocephalus, microcephaly, dolichocephaly, chorioretinitis, optic nerve atrophy, retinal colobomas, microphtalmia, blindness, hearing loss, developmental delay, pyschomotor retardation, spastic quadriplegia, seizures, heart abnormalities, ataxia, fetal hydrops, cataracts	CT: periventricular and intracranial calcifications, diffuse and periventricular brain substance loss, gyral malformations, shizencephaly, cerebellar hypoplasia, calcification of the lens MRI: ventriculomegaly, cerebral atrophy, corpus callosum atrophy and agenesis, encephalomalacia, cerebellar hypoplasia, intracranial hemorrhage, periventricular cysts	[12,13,67,71,73,74,75,76,77,78,79,80,81,82,83]

* ND (no data).

**Table 3:** Selected recent contributions of LCMV mouse model to immunological research.

Immune Responses	Areas of Immunological Research with a Major Contribution of LCMV Mouse Experimental Model
Innate immunity	
Recognition of pathogen-associated molecular patterns by pattern-recognition receptors	Recognition of LCMV single-stranded RNA by TLR-7 and -8; recognition of double-stranded RNA and 5′-triphosphate RNA that are synthesized during the LCMV replication cycle by MDA-5 and RIG-I; role of TLR-2 in the immune response to LCMV
Innate immunity signal-transduction pathways and transcription factors	Signaling pathways leading to the activation of transcription factors IRF-3, AP-1 and NF-κB that induce the synthesis of IFN-β, IFN-α4 and other pro-inflammatory cytokines
Biology of type I IFNs	Role of interferons in regulating the activity of innate immune cells; differential regulation of interferon-stimulated genes during infection with various LCMV strains; relative contribution of STAT1 on innate and adaptive immunity during LCMV infection; role of IFN-mediated signals in CD8+ T-cell responses
The role of NK-cells in the pathogenesis of viral infections	Cytolytic effect of NK-cells on activated CD4+ and CD8+ T-cells in viral infection; Treg, Th17 and Th2 cells are more sensitive to lysis by LCMV-induced activated NK-cells
Specific immunity	
Thymic depletion in chronic viral infections	Chronic LCMV infection induces severe thymic depletion, mediated by CD8+ T cell-intrinsic type I IFNs and STAT-2 signaling pathway
Viral infection as a trigger of Treg cell impairment and associated immune-mediated pathology in autoimmunity	LCMV infection leads to the loss of IFN type I-dependent Treg cells, which is subsequently compensated by the conversion of Vβ5+ conventional T cells into iTreg cells; delayed replenishment of Treg cells in Vβ5-deficient mice compromises suppression of microbiota-dependent activation of CD8+ T-cells leading to the development of colitis
Metabolic alterations in the liver during chronic viral infections	Type I interferon-mediated suppression of the hepatic urea cycle and subsequent suppression of virus-specific CD8+ T-cell responses and ameliorated liver pathology
Indirect protective role of non-neutralizing antibodies in viral infections	LCMV-specific monoclonal Abs can prevent the establishment of chronic infection in an Fc-receptor-independent manner by inducing the differentiation of Ly6Chi inflammatory monocytes into antigen-presenting cells leading to an early activation of virus-specific CD8+ T-cells

Toll-like receptors (TLR), melanoma differentiation-associated protein (MDA)-5, retinoic acid-inducible gene-I-like receptors (RIG-I), interferon-response factor (IRF), signal transducer and activator of transcription (STAT).
